# Unmet Information Needs of Patients with Rheumatic Diseases: Results of a Cross-Sectional Online Survey Study in Germany

**DOI:** 10.3390/ijerph19127071

**Published:** 2022-06-09

**Authors:** Christian Becker, Matthias Diener, Axel J. Hueber, Jörg Henes, Martin Krusche, Yuriy Ignatyev, Susann May, Ulrike Erstling, Corinna Elling-Audersch, Johannes Knitza, Felix Muehlensiepen

**Affiliations:** 1 Digital Rheuma Lab, 10823 Berlin, Germany; christian.becker@digitalrheumalab.de (C.B.); matthias.diener@digitalrheumalab.de (M.D.); 2 Division of Rheumatology, Klinikum Nürnberg, Paracelsus Medical University, 90419 Nürnberg, Germany; axel.hueber@klinikum-nuernberg.de; 3 Center for Interdisciplinary Clinical Immunology, Rheumatology and Auto-Inflammatory Diseases and Department of Internal Medicine II (Oncology, Hematology, Immunology and Rheumatology), University Hospital Tübingen, 72076 Tübingen, Germany; joerg.henes@med.uni-tuebingen.de; 4 Department of Internal Medicine III, University Medical Center Hamburg-Eppendorf, 20251 Hamburg, Germany; m.krusche@uke.de; 5 Center for Health Services Research, Faculty of Health Sciences Brandenburg, Brandenburg Medical School, 15562 Rüdersdorf bei Berlin, Germany; yuriy.ignatyev@mhb-fontane.de (Y.I.); susann.may@mhb-fontane.de (S.M.); 6 Fachverband Rheumatologische Fachassistenz e.V., 51465 Bergisch Gladbach, Germany; u.erstling@forum-rheumanum.de; 7 Deutsche Rheuma-Liga Bundesverband e.V., 53111 Bonn, Germany; elling@rheuma-liga.de; 8 Department of Internal Medicine 3, Friedrich-Alexander-University Erlangen-Nürnberg and Universitätsklinikum Erlangen, 91057 Erlangen, Germany; johannes.knitza@uk-erlangen.de

**Keywords:** chronic disease, rheumatology, telemedicine, eHealth, mHealth, patient perspective, mixed-methods, qualitative research, survey

## Abstract

To effectively self-manage a chronic disease, patients require specific education. In clinical routines, rheumatologists and other healthcare professionals often cannot devote the necessary time to adequately educate their patients. Digital technologies such as mobile applications represent promising tools to overcome this problem. This study aims to identify unmet information needs of patients with rheumatic diseases to inform the conception of a mobile education application. An online national survey was developed together with rheumatic patients and rheumatologists and distributed between June and September 2021 via social media (Instagram, Facebook, Twitter), QR code and email. Self-reported rheumatic patients, rheumatologists, specialized rheumatology nurses (SRN) and self-reported relatives of rheumatic patients were eligible to participate in the survey. Three major topics were addressed: (1) How well do patients feel informed about disease-relevant topics; (2) how important do patients rate different disease-relevant topics; and (3) patient willingness to adopt digital education services. Responses of 254 patients and 53 SRN were analyzed. Most patients were female (91%; *n* = 231), the median age was 48 years and the most common disease was rheumatoid arthritis (23%; *n* = 59). Only 24% of patients perceived their disease education level as very good or good compared to an SRN estimate of 42%. The three information topics rated as most important (very/important) were: individual disease (98%), medication (94%) and coping techniques (91%). In total, 89% of patients asserted that they would very likely, likely or rather likely use digital education tools in the future to learn about their condition, and 82% of SRN would very likely, likely or rather likely recommend digital information services to their patients. These findings depict currently unmet patient information needs and a high willingness of patients and SRN to use digital education services. A mobile education application is currently adapted based on these results and will be evaluated in a multicenter study.

## 1. Introduction

Rheumatic and musculoskeletal diseases (RMD) are a group of heterogeneous chronic diseases with different respective symptoms, disease courses and prognoses. Patient education [1] is crucial to enable optimal self-management [2] and overall disease management [3]. Increasing evidence [1] highlights the benefits of patient education, such as enhanced adherence to pharmacological treatment [4,5] and improved coping skills, as well as physical and psychological health status [6,7].

Especially newly diagnosed patients are facing a lot of distress and new questions [8,9]. Furthermore, patients with established diagnosis [10] are confronted with questions regarding management of disease flares, infections or medication side effects. Access to rheumatologists is becoming increasingly more difficult as a diminishing number of rheumatologists [11] are facing increasing patients demands. Currently, this has resulted in a significant diagnostic delay of months in Germany [12] and very short appointment lengths of typically 15 min per patient [13], which prevents the crucial provision of comprehensive information and education to RMD patients [14].

Patient education can be delivered through a variety of modes, including individual [15] or group [6] face-to-face-meetings or simple leaflets [16]. Digital formats promise improved delivery of personalized information with instant and permanent access, irrespective of time and place. Widespread usage of smartphones among RMD patients [17], as well as increasing usage of digital information [18], promises feasible implementation into clinical routine. Disease-related information has repeatedly [17,18] been identified by RMD patients as the most important feature in potential mobile applications. First randomized controlled trials (RCT) evaluating digital patient education reported improved self-efficacy [19], quality of life [19] and overall health status [20].

The aim of this study was to investigate, through an online survey distributed via social media (Instagram, Facebook, Twitter), QR code and email, (1) currently unmet information needs and (2) preferences of RMD patients in the use of digital educational services informing the creation of an educational mobile application for RMD patients.

## 2. Materials and Methods

The online survey was created by rheumatologists and rheumatic patients in cooperation with the Berlin-based start-up Digital Rheuma Lab. Digital Rheuma Lab consists of a six-person team of patients, business economists, psychologists and software developers in Berlin who aim to develop digital solutions for rheumatology care (https://www.digitalrheumalab.de/, accessed date: 4 June 2022). (1) Patients with self-reported RMD, (2) their relatives, (3) specialized rheumatology nurses (SRN) and (4) rheumatology specialists (rheumatologists) were identified as the most important stakeholders in the context of patient education and defined as respective target groups of the survey. Subsequently, three central areas of interest to be investigated were defined.

1. Status quo: How do patients feel educated about their disease and its accompanying circumstances at the time of the survey?
2. Key topics: Which topics are particularly important when dealing with and coping with the disease, and how well do patients feel informed about these topics?
3. Willingness to adopt digital services in the future: How do patients rate their willingness to adopt digital services in the future; what potentials and what risks do they perceive in this context?

The survey additionally investigated how often the pre-established topics are addressed in day-to-day routine of SRN and patients. To achieve this, SRNs were asked to provide an estimate of how often questions about each topic area were directed to them by patients. To put that into the context of increasing digitization in the field of rheumatology and healthcare in general, also the willingness to use or recommend digital solutions to increase disease knowledge was examined for patients and SRN.

Four questionnaires in the German language, each adapted slightly to the specific target group, were created and integrated into a coherent document in Google Forms. First, participants were asked which target group they belonged to, with their response determining the following specific version of the questionnaire. The patient questionnaire contained 28 questions and the SRN questionnaire contained 25 questions. Participants were able to choose predefined answers in multiple choice fields or assess closed questions via a 6-point Likert scale. The pre-defined answer options were derived from the results of unpublished, preceding pilot-interviews with individual patients (*n* = 12) and structured workshops (*n* = 3) with an average participation of 6 patients to allow a qualified preselection of response options. To also reflect the interest in acquiring additional insight into the fields of interest mentioned previously, open-ended response fields were integrated into the questionnaires. Free-form responses were added to contextualize and deepen the multiple-choice responses. Demographic as well as psychographic information were asked at the end of each questionnaire. The survey was made available online from 01.06.2021 to 30.09.2021. Its distribution was online via social media (Twitter, LinkedIn, Instagram, Facebook) and email, along with flyers displaying QR codes in the clinical settings of Berlin Charité, Leipzig University Hospital, Tübingen University Hospital, and Darmstadt Hospital. All German-speaking patients with self-reported RMDs, rheumatologists, SRN and self-reported relatives of RMD patients were eligible. All participants provided consent. Individuals not proficient in German language were excluded. Apart from this, no exclusion criteria applied. 

Socio-demographic and clinical characteristics of the sample were assessed using descriptive statistics. In order to analyze proportions of use/recommendations of information sources by patients and SRN, as well as perceived benefits and disadvantages of digital solutions for patient education, χ2-tests with continuity correction were used. Diagnostic categories were analyzed on the level of main diagnoses. To allow comparison between different diagnostic categories, all diagnoses were attributed to four overall groups: (1) arthritis condition; (2) connective tissue disease; (3) fibromyalgia; (4) others. In order to examine the differences between ordinal data (perceived education levels and satisfaction with the currently available information offering of patients and SRN as well as willingness to use/recommend digital solutions to increase disease knowledge of patients and SRN), Wilcoxon rank sum test with continuity correction and Kruskal–Wallis test were used. To compare Kruskal-type ranked data, we used Conover’s non-parametric all-pairs comparison test. The information needs of patients were examined using correlations analysis (Spearman’s rank correlation coefficients) of importance and self-perceived education level relating different topics. The negative correlations between importance and status quo of perceived education levels were assumed as a sign of unmet Information needs. The calculation of Spearman’s rho was supplemented by the visual check of scatterplots. The level of significance was set at 0.05. Descriptive analysis was conducted using statistical packages SPSS 23. The Conover’s non-parametric all-pairs comparison test was carried out with the package PMCMRplus running in R version 1.9.4 (R Core Team, 2017).

The study was conducted in compliance with current data protection regulations and the Helsinki Declaration in its current form. All study participants were informed about the research project. Participation and submission of the questionnaire were considered as consent to study participation. Due to the non-interventional, anonymous nature of the study and aim of improving the current service quality, no ethical approval was required. Reporting of the study methods and results was carried out according to the Checklist for Reporting Results of Internet E-Surveys [21].

## 3. Results

From June to September 2021, a national online-based survey study on the status quo of patient education with the focus on patients (*n* = 254) and SRN (*n* = 53) was conducted. Due to the small number of participants and the corresponding limited significance of the results, relatives (*n* = 7) and rheumatologists (*n* = 6) were not considered in the further analysis. Most participating patients (91%; *n* = 231) were female. Various RMD were reported by participating patients, with rheumatoid arthritis (25%; *n* = 64), SLE (21%; *n* = 53), psoriatic arthritis (11%; *n* = 29) sjögren syndrome (9%; *n* = 24), ankylosing spondylitis (8%; *n* = 21) and fibromyalgia (4%; *n* = 10) as the most frequent indications across the survey population. Participants were a median of 48 years old (mean: 45 years) and had a rheumatic disease for a median of 5 years (mean: 17 years) (Table 1).

All participating SRN were female and predominantly employed in the outpatient setting (96%; *n* = 51 compared to 4%; *n* = 2 in the clinical setting). Participants from the SRN cohort were a median of 51 years old (mean: 46 years) and had been in their profession for a median of 7 years (mean: 10.5 years) (Table 2).

### 3.1. Status Quo of Perceived Education

In total, 307 participants were surveyed (Table 3) across the groups of patients (*n* = 254) and SRN (*n* = 53) with the intent to establish an understanding about the perceived education levels in the mentioned groups as well as their respective satisfaction with the currently available information offering.

On a scale of 1 (insufficient) to 6 (very good), only 24% of patients (*n* = 62) described their education level about their disease as very good (6) or good (5), approximately half (54%; *n* = 136) as satisfactory (4) or sufficient (3), while 22% (*n* = 56) felt deficiently (2) or insufficiently (1) educated (Table 3).

In contrast to that, 42% (*n* = 22) of the participating SRN considered the education level of their patients to be very good (6) or good (5), and just over half of the responders (51%; *n* = 27) assessed the education level to be satisfactory (4) or sufficient (3). A minority perceived the level of education to be deficient (2) or insufficient (1).

Patients reported doctor consultation (80%; *n* = 203), search engines and internet research (e.g., Google) (70%, *n* = 177) and online forums (65%; *n* = 166) as the top 3 most prevalent sources of information. Excluding the face-to-face interaction with the rheumatologist, patients’ consultation of offline information sources, such as in-person seminars (8%; *n* = 20), paper brochures (41%; *n* = 105) or self-help groups (32%; *n* = 82), is notably less frequent compared to online information sources.

**Figure 1:**
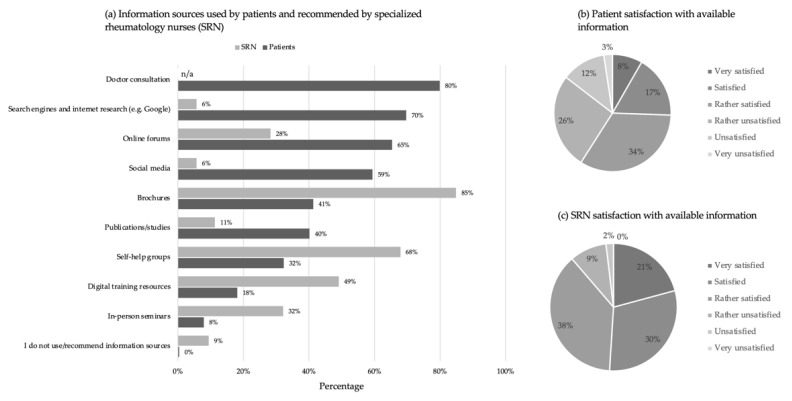
Information sources used by patients and recommended by specialized rheumatology nurses (SRN) and satisfaction with the currently available information. (**a**–**c**) Data is displayed as the percentage of total patients and SRN that responded to the question.

Regarding the types of information sources SRN recommend to patients to learn about their disease, 85% (*n* = 45) of respondents mentioned paper brochures, 68% (*n* = 36) self-help groups, 49% (*n* = 26) digital education resources, 32% (*n* = 17) in-person seminars, 28% (*n* = 15) online forums, 11% (*n* = 6) publications and studies, 6% (*n* = 3) social media and 6% (*n* = 3) online search engines. 9% (*n* = 5) also reported recommending no information (Figure 1a).

Although most patients use different information sources frequently, just over a quarter of the surveyed patients (26%; *n* = 65) mentioned that they felt very satisfied or satisfied with the available information. Another 60% (*n* = 152) reported to feel rather satisfied or rather unsatisfied while 15% (*n* = 37) expressed their dissatisfaction (Figure 1b). In contrast to that, a large majority of 89% (*n* = 47) of the surveyed SRN were very satisfied (6), satisfied (5) or rather satisfied (4) with the information available to patients and just over 10% were dissatisfied (Figure 1c).

### 3.2. Relative Importance of Respective Disease Topics by Patients

**Figure 2:**
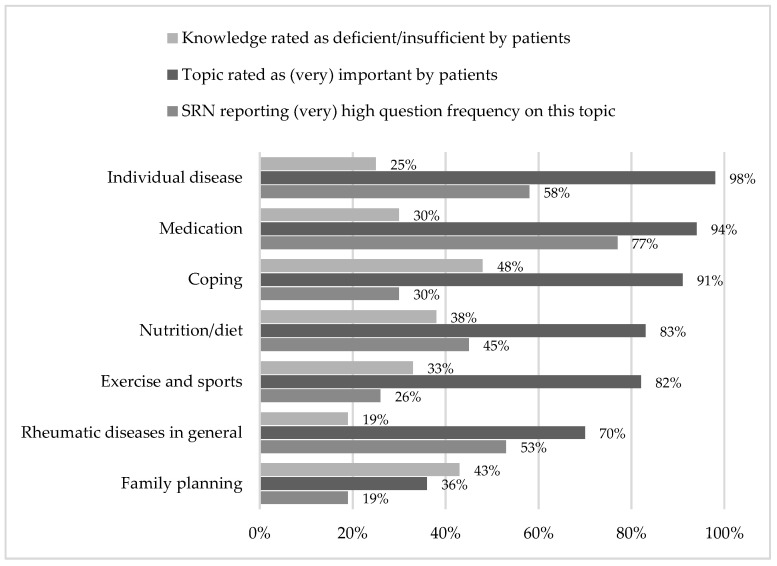
Unmet needs in patient education based on patient-rated topic importance, self-rated education level and SRN-reported question frequency in daily care routine.

Building on the initial questions on overall perceived education levels, as well as the levels of use and recommendation of a selected range of information sources, a set of pre-established topics were scrutinized, i.e., their relative importance for patients, their self-perceived education level and the frequency of patient questions regarding respective disease topics (Figure 2).

Addressing the importance of information about their specific disease, a vast majority of all surveyed patients (98%; *n* = 248) attributed high importance to the topic. Similarly, patients attributed high relevance to the topic of medication, with 94% (*n* = 239) answering that information on medication is (very) important and just above 5% (*n* = 15) stating that the topic is of lesser importance. 

Disease coping also appeared to be critical. Patients asserted with 91% (*n* = 230) of all respondents that the topic was of the utmost importance. The importance to patients became increasingly clear, as only 3% (*n* = 7) of all surveyed patients indicated that the topic for them was unimportant. Additionally, patients rated the topics of nutrition and exercise with very similar results. Just over 80% stated that information on the two matters was (very) important.

Having highlighted the topics with higher patient-perceived importance, it is also necessary to address information on family planning and rheumatic diseases, in general, as matters rated with comparatively lower relevance. Whereas information on the individual disease yielded a very clear result, patients rated that the importance of information on rheumatic diseases had greater distribution. While a majority (70%; *n* = 179) asserted that this was a (very) important point of consideration, approximately one-third also rated the topic with lower relevance. Asked how important information on the topic of family planning in rheumatic diseases was to them, only 36% (*n* = 92) answered (very) important and the majority (64%; *n* = 162) indicated that the issue was of lower relevance.

### 3.3. Self-Perceived Knowledge Regarding Respective Disease Topics

In addition to addressing the relative importance of the mentioned disease topics to patients, the survey also yielded results on the patient-perceived knowledge regarding the respective subjects. Patients rated their knowledge on disease coping as lowest of all proposed subjects, with 48% (*n* = 123) indicating to have deficient or insufficient knowledge on the matter. Although patients reported that information on family planning with rheumatic diseases is in comparison less relevant and the surveyed patient population was predominantly female, 43% (*n* = 109) stated that they had little knowledge on the topic. The survey results on diet (38%; *n* = 97), exercise and sports (33%; *n* = 85) and medication (30%; *n* = 76) yielded similar results, although the knowledge deficit appears to be more apparent for lifestyle themes (diet and exercise) than for medication. Although information on the individual disease was the subject that patients rated with the highest relevance of all with 98% (*n* = 248) (Figure 2), a quarter of all surveyed patients still felt deficiently or insufficiently informed on the topic. In contrast to that, patients felt best informed on the topic of rheumatic diseases in general, with 19% (*n* = 48) expressing a lack of knowledge.

### 3.4. Frequency of Patients Questions Regarding Respective Disease Topics

The results show that with questions about medication (77%; *n* = 41), the individual disease (58%; *n* = 31) or rheumatic diseases in general (53%; *n* = 28), which are directly related to the therapy and the care situation, are increasingly asked by patients in everyday care. In contrast, however, the survey data also show that accompanying therapy topics are addressed less frequently in everyday practice. For instance, SRNs estimate the frequency of questions on the topic of coping to be only 30% (*n* = 16) (very) high. It’s clear that the topic has very high relevance (91%; *n* = 230) and at the same time about half of the patients surveyed state that they have little to no knowledge regarding this topic.

Similarly, only a quarter of SRN reported that the topic of exercise and sport is (very) frequently addressed by patients, although patients by a substantial majority attribute high importance to the topic. Regarding nutrition, SRNs reported a (very) high frequency in everyday care. In correspondence with patients attributing the lowest relevance of all pre-established topics to the subject of family planning, SRNs also reported the lowest frequency (19%; *n* = 10).

### 3.5. Willingness to Use Digital Tools to Increase Disease Knowledge

**Figure 3:**
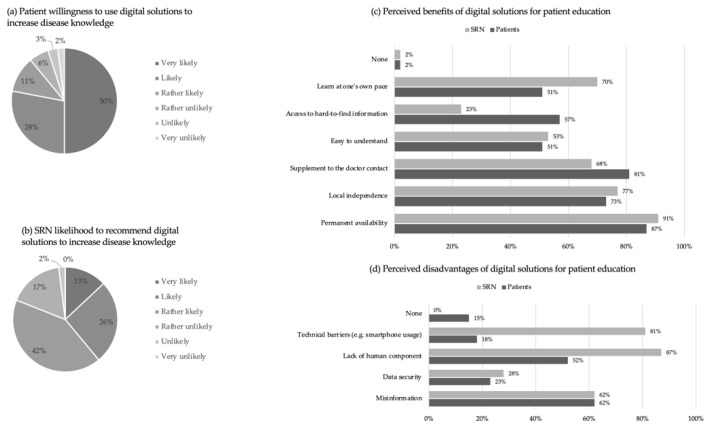
Patient and SRN willingness to use/recommend digital solutions to increase disease knowledge with perceived benefits and disadvantages. (**a**–**d**) Data is displayed as the percentage of total patients and SRN that responded to the question.

When asked how likely they were to use digital offerings to learn about their condition in the future, a significant majority of patients (89%; *n* = 226) asserted that they were very likely, likely or rather likely to do so. The minority of surveyed patients (11%; *n* = 27) stated they were rather unlikely, unlikely or very unlikely to utilize digital solutions (Figure 3a).

In response to whether they would recommend digital information services to their patients in the future, 40% (*n* = 21) of the participating SRN reported they were very likely or likely to do so. Another 42% (*n* = 22) considered this rather likely. Moreover, 17% (*n* = 9) felt it was rather unlikely and one participant (2%) said it was unlikely to recommend digital information services in the future (Figure 3b).

Addressing the question of what advantages they would associate with digital education resources, 87% (*n* = 221) named permanent availability, 81% (*n* = 207) asserted a complement to physician contact, 73% (*n* = 185) mentioned independence of location, 57% (*n* = 145) noted the access to hard-to-find information and 51% (*n* = 129) discussed the possibility of self-paced learning, as well as the ease of understanding information. Further, 2% (*n* = 6) did not associate any advantages with digital solutions. For the same question, 91% (*n* = 48) of SRN specified permanent availability, 77% (*n* = 41) noted locational flexibility, 70% (*n* = 37) said self-paced learning, 68% (*n* = 36) rated the complement to physician contact as a benefit, 53% (*n* = 28) mentioned the ease of understanding content and 23% (*n* = 12) asserted the access to hard-to-find information as positive. One participating rheumatology assistant did not see any advantages (Figure 3c). 

When asked what disadvantages and risks patients saw in digital education offers, 62% (*n* = 157) mentioned the risk of misinformation. Approximately half of the participating patients (52%; *n* = 133) regarded the lack of the human element in digital solutions as a downside while concerns for data security (23%; *n* = 59) or technological barriers (18%; *n* = 45) were also expressed. Additionally, 15% (*n* = 37) perceived no disadvantages at all. Similarly, 87% (*n* = 46) of SRN expressed the lack of a human component as the main downside. In addition, 81% (*n* = 43) noted technological barriers, 62% (*n* = 33) stated misinformation, and 28% (*n* = 15) expressed concerns about data security as disadvantages (Figure 3d).

### 3.6. Statistical Analysis: Differences between the Sub-Groups

There were significant differences of perceived education level between four diagnostic group (Table 4). However, after the application of methods to counteract the multiple comparisons problem in the course of pairwise comparisons, the differences could not be confirmed.

SRN reported a better overall perceived education level than patients (4.24 ± 1.11 vs. 3.59 ± 1.35, W = 4750, *p* < 0.001). In addition, SRN were more satisfied with the currently available information (4.58 ± 0.99 vs. 3.76 ± 1.19, W = 4103.5, *p* < 0.0001). However, patients would more like to use digital solutions to increase disease knowledge than SRN would it recommend (5.10 ± 1.21 vs. 4.32 ± 0.98 W = 9747.5, *p* < 0.0001). Table 5 shows differences between patients and SRN that relate to several information sources which show perceived benefits and disadvantages of digital solutions.

The correlations analysis revealed unmet information needs of patients related to certain topics. These fields were nutrition/diet (rho= −0.19, *p* < 0.01), coping (rho= −0.19, *p* < 0.01) and exercise and sports (rho= −0.18, *p* < 0.01). While the information needs other topics to be considered ‘unmet, but they did not reach statistical significance. A single exception was the topic of family; the need seemed to be covered (rho= 0.13, *p* < 0.05). However, the interest in this topic was low.

## 4. Discussion

This questionnaire study reports on information needs and willingness to use digital educational services regarding RMD patients. To our knowledge, this questionnaire study is the first to compare patient perceived information needs to the needs perceived by caring health care professionals. Our results revealed that SRN consider patients to be better informed than patients themselves. Most patients rated disease-related information to be highly important, whereas only a quarter of patients felt well or very-well informed. Currently most prevalent unmet information needs, determined by the gap between perceived relevance and self-perceived knowledge on the subject, are coping with the disease followed by medication, information on the individual disease and nutrition/diet. Regarding information sources the discrepancy in perception between SRN and patients persisted. SRN rated traditional information sources such as brochures as more important and underestimated the importance of digital information sources such as social media and online search engines. Similarly, SRN were more satisfied with the information currently offered compared to patients.

The majority of SRN would recommend digital tools to patients in the future and similarly patients reported a high willingness to use digital information services. The main advantage of digital information resources reported by patients was the advantage of constant availability of information, followed by valuable addition to in-person physician contact and gained independence. Fear of misinformation, specifically about choosing the appropriate information service, as well as fear of losing physical face-to-face contact to the physician are key drawbacks, reported by patients. Correspondingly, SRN reported technical barriers and lack of human contact as major barriers, specifically in regard to older patients. 

The results are in line with a previous survey [17] that depicted the internet as the most frequently accessed source of information for RMD patients. This survey also revealed the need to help patients while navigating available online information, as the mean eHealth literacy was relatively low. The reported concerns regarding digital services, particularly in respect of the risk of loss of human contact in medical treatment, are in line with the results of a recent mixed-methods study on telemedicine in rheumatology [22]. The authors propose individual assessment and scalable telemedicine care concepts to address concerns and ensure close physician-patient relationship. mHealth and digital educational services targeting information needs and patient education ought to be a part of it. According to our findings, these might be particularly effective if they focus on coping with the disease followed by medication, information on the individual disease and nutrition/diet. SRN work force already is indispensable, and its relevance and responsibility will likely increase even further [23]. In both standard and future care, health care professionals should be aware of the information needs of their patients and actively support them with adequate resources. Therefore, for health care professionals, there is a need for further training in this area. In addition, our results offer reason to review the information currently provided to patients. Clinical study participant information is typically provided in written form, which is often difficult for patients to read and understand. More interactive formats, like videos, could help communicate this important information [1,17,24].

There are a few limitations to our study. Initially the survey addressed rheumatologists and RMD-patients’ relatives. Response rates in those two cohorts were too low and were thus excluded from further analysis. We attribute the low response rate among rheumatologists to the high clinical burden and scarce time resources during COVID-19. Thus, we are considering a follow-up with physicians and other stakeholders, such as physiotherapists and occupational therapists. As the study was conducted via an online survey, a selection bias is very likely. This is reflected by the rather young RMD population with a mean age of 45. Moreover, not all RMD patients were equally represented, as, for example, the proportion of SLE patients in the study population was relatively high. In contrast, the questionnaire was not answered by any patients suffering osteoarthritis. Additionally, the sampling method will most likely have led to the exclusion of individuals with low digital literacy, Finally, almost exclusively women participated in our survey, which further limits generalizability.

## 5. Conclusions

Our results highlight currently unmet RMD patient information needs and the willingness of patients and SRN to use respective digital services. Based on these results, we are developing a dedicated digital service, which will be evaluated in a multicenter study.

## Figures and Tables

**Table 1:** Patient characteristics (mean or *n* (%)).

	Patients *n* = 254 (100%)
Age, years	45 (Median = 48; Range = 15–74)
<30	50 (20%)
31–40	47 (19%)
41–50	59 (23%)
51–60	76 (30%)
>60	22 (9%)
Women	231 (91%)
Diagnosis	
Rheumatoid arthritis	64 (25%)
Systemic lupus erythematosus	53 (21%)
Psoriatic arthritis	29 (11%)
Ankylosing spondylitis	20 (8%)
Sjögren syndrome	24 (9%)
Fibromyalgia	10 (4%)
Other	53 (21%)
Years since diagnosis	17 (Median = 5; Range = 0–34)

**Table 2:** Characteristics of SRN (mean or *n* (%)).

	SRN *n* = 53 (100%)
Women	53 (100%)
Age, years	46 (Median = 51; Range = 22–63)
<30	8 (15%)
31–40	9 (17%)
41–50	8 (15%)
51–60	23 (43%)
>60	5 (9%)
Working place	
Outpatient setting	51 (96%)
Clinical setting	2 (4%)
Working practice in years	10.5 (Median = 7; Range = 0–40)

**Table 3:** Results—patients’ compared to SRN-perceived education levels and satisfaction with the currently available information offering.

	All Participants *n* = 307		Patients *n* = 254		SRN *n* = 53	
	*n*	%	*n*	%	*n*	%
**Overall perceived education level**						
Very good	31	10%	25	10%	6	11%
Good	53	17%	37	15%	16	30%
Satisfactory	95	31%	74	29%	31	40%
Sufficient	68	22%	62	24%	6	11%
Deficient	42	14%	39	15%	3	6%
Insufficient	18	6%	17	7%	1	2%
**Satisfaction with the currently available information**						
Very satisfied	32	10%	21	8%	11	21%
Satisfied	60	20%	44	17%	16	30%
Rather satisfied	105	34%	85	33%	20	38%
Rather unsatisfied	72	23%	67	26%	5	9%
Unsatisfied	32	10%	31	12%	1	2%
Very unsatisfied	6	2%	6	2%	0	0%
Questions: “How well do you feel educated about your condition and its accompanying circumstances?“; “How satisfied are you with the information available to you?“						

**Table 4:** Perceived education level and satisfactions with available information by diagnostic group.

	Overall Diagnostic Group								Statistics	
	AC *n* = 114		CTD *n* = 79		F *n* = 10		Other *n* = 51		H df = 3	*p*-Value
	M	SD	M	SD	M	SD	M	SD		
Perceived education level	3.88	1.23	3.41	1.49	3.1	0.88	3.33	1.38	9.99	*p* < 0.05
Satisfaction with available information	3.87	1.21	3.56	1.24	3.7	0.95	3.84	1.1	2.85	NS

AC = arthritis condition; CTD = connective tissue disease; F = fibromyalgia; Other = other diagnoses; NS = not significant.

**Table 5:** Information sources, perceived benefits and perceived disadvantages of digital solutions by participant group.

	Patients *n* = 254		SRN *n* = 53		Chi-Square df = 1	*p*-Value
	*n*	%	*n*	%		
**Information sources**						
Doctor consultation	203	80%	n/a*	n/a *	120.45	<0.0001
Paper brochures	105	41%	45	85%	31.59	<0.0001
Online forums	166	65%	15	28%	23.37	<0.0001
Social media	151	59%	3	6%	48.62	<0.0001
Publications/studies	102	40%	6	11%	14.75	<0.0001
Search engines and internet research	177	70%	3	6%	71.49	<0.0001
In-person seminars	20	8%	17	32%	22.00	<0.0001
Self-help groups	82	32%	36	68%	22.06	<0.0001
Digital training resources	46	18%	26	49%	7.69	<0.0001
I don’t use/recommend information sources	1	0.4%	5	9%	14.28	<0.0001
**Perceived benefits**						
Permanent availability	221	87%	48	91%	0.24	NS
Independence of location	185	73%	41	77%	0.26	NS
Complement to physician contact	207	81%	36	68%	4.11	<0.05
Ease of understanding information	129	51%	28	53%	0.01	NS
Access to hard-to-find information	145	57%	12	23%	19.47	<0.0001
Possibility of self-paced learning	129	51%	37	70%	5.67	=0.01
None	6	2%	1	2%	0.09	
**Perceived disadvantages**						
Risk of misinformation	157	62%	33	62%	0.01	NS
Data security	59	23%	15	28%	0.37	NS
Lack of human element	133	52%	46	87%	19.99	<0.0001
Technological barriers	45	18%	43	81%	83.17	<0.0001
None	37	15%	0	0%	7.46	<0.01

* In alignment with the “Fachverband Rheumatologische Fachassistenz e.V” (German SRN association) it has been decided to not include the option “Doctor consultation” in the questionnaire that was presented to participating SRN as SRN already work in the context on doctor consultation; NS = not significant.

## Data Availability

Data are available on reasonable request. The data will be shared if there is a reasonable request for it.
